# Implementing a ‘Lead [Apron]-Free’ Cardiac Catheterization: Current Status

**DOI:** 10.1007/s11886-024-02102-w

**Published:** 2024-07-25

**Authors:** Akash H. Patel, Vishal Patel, Yicheng Tang, Sai Shah, George Tang, Morton J. Kern

**Affiliations:** 1 Division of Cardiology, University of California, Irvine, California USA; 2 Division of Internal Medicine, University of California, Irvine, California USA; 3 Division of Cardiology, University of California – Irvine and VA Long Beach, Long Beach, California USA

**Keywords:** Radiation Safety, Lead-Free, Cardiac Catheterization

## Abstract

**Purpose of Review:**

In this review, we discuss the status of novel radiation shielding and other methods to reduce radiation exposure and its associated health risks within the CCL.

**Recent Findings:**

There are many devices on the market each with its unique advantages and inherent flaws. Several are available for widespread use with promising data, while others still in development.

**Summary:**

The field of percutaneous transcatheter interventions includes complex procedures often involving significant radiation exposure. Increased radiation exposes the proceduralist and CCL staff to potential harm from both direct effects of radiation but also from the ergonomic consequences of daily use of heavy personal protective equipment. Here we discuss several innovative efforts to reduce both radiation exposure and orthopedic injury within the CCL that are available, leading to a safer daily routine in a “lead [apron]-free” environment.

## Introduction

As the field of percutaneous transcatheter interventions continues to include more complex procedures often requiring significantly more radiation exposure time. These procedures include but are not limited to multi vessel percutaneous coronary intervention (PCI), complex peripheral interventions, high risk-PCI, chronic total occlusion (CTO), transcatheter aortic valve replacement, and transcatheter edge-to-edge repair of the mitral and tricuspid valves. Continuing interest in cath lab safety has focused on various health hazards from cataracts to cancer working in a fluoroscopic laboratory. [1–3]. Thyroid cancer and a disproportionate incidence of left-sided brain tumors (more than half being glioblastoma multiforme) have been reported among interventional cardiologists [4]. When discussing the cumulative detrimental health effects of working in a CCL, orthopedic injury (particularly those related to the lumbar and cervical spine) from heavy leaded aprons are often considered as collateral occupational damage [5, 6]. As the volume of complex procedures increases, physical stress associated with procedural performance will exacerbate the degree and prevalence of orthopedic injuries. For this reason alone, there are several innovative efforts aiming to reduce both radiation exposure and orthopedic injury within the CCL. This initiative, moving towards a “lead-free” environment, extends beyond increases in personal protective equipment and includes enhanced surrounding patient shielding at the table, partial room shielding, suspended radiation protection systems (SRPS), enclosed and remote operator shielding, and enhanced tube shielding and patient drape shields. This manuscript is designed to address current lead-free or lead-equivalent devices on the market and to give the reader a broad perspective on the innovative solutions to contain and reduce radiation scatter.

## Patient Shielding at the table – The Eggnest XR

**Fig. 1:**
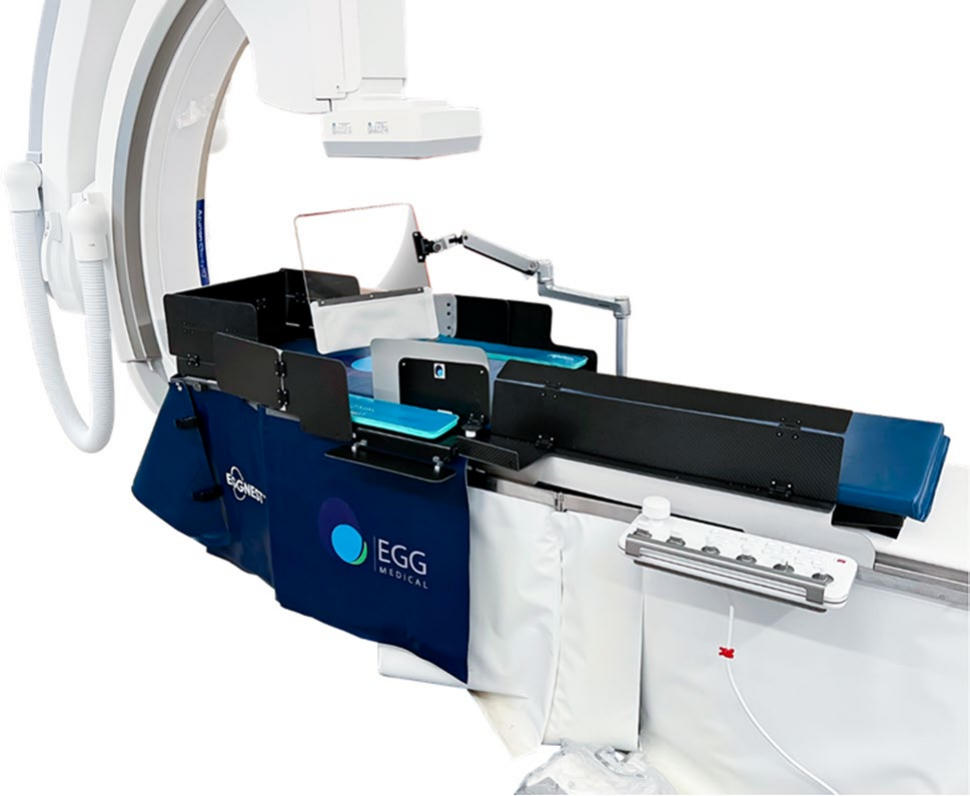
The EggNest XR system is comprised of a carbon-fiber sled base and mattress, with built in CPR board, as well as shields positioned between the operator and patient to mitigate scatter radiation exposure with 0.5 mm lead-equivalent. (*Reprinted with permission from EggNest XR Medical)*

The EggNest XR System (Egg Medical (R)) is the second-generation radiation protection system developed on the Eggnest Protect platform. Developed by CEO and interventional cardiologist Dr. Robert Wilson, the system focuses on patient scattered radiation reduction. The EggNest XR system (Fig. 1) is comprised of a carbon-fiber sled base and mattress, with built in rigid but radiotranslucent CPR board, as well as shields positioned between the operator and patient to mitigate scatter radiation exposure with 0.5mm lead-equivalent. The key components of the EggNest XR system can be attached or replaced in a modular fashion with existing fluoroscopy systems, providing ease of application and use. Limitations include inability to be used in biplane systems or for neurointerventional cases. Leaded garments, including aprons, thyroid shields, and glasses still need to be worn. These limitations are highlighted in Table 1. The effectiveness of the EggNest system has been validated by studies done measuring scatter radiation at six key positions around the table in the five most common projections, resulting in a reduction of 88% to 94% of the total room scatter radiation [7]. Initial studies reported over 91% overall reduction. Investigations on scatter radiation report multiorgan effect upon the operator and cardiac catheterization laboratory personnel, noting sixfold increased risk in cataracts, threefold increased risk of skin, thyroid, and brain cancer, and twice the risk of cardiovascular disease compared to controls. [8]

**Table 1:** This comprehensive summary table displays all eight current devices on the market to try and limit radiation exposure to the operator and cath lab staff.*

	Device Features	Pro’s	Con’s
EggNest XR	- Carbon-fiber sled base and mattress - Built in CPR board	- Ability to add to existing fluoroscopy systems (modular design), minimizing overhaul need	- Leaded garments still required - Cannot be used for biplane systems - Cannot be used for neurointerventional cases
PROTEGO	- Upper shield mounted on a spring arm with magnets for quick deployment and release - Two patient visualization screens with cameras - Arm board built in radiation drapes	- Allows for unimpeded C-arm motion - Quick deployment and release mechanism - Left/Right Cameras for constant patient visualization	- Floor or ceiling space required for mounting mechanism - Adds 100lbs reducing maximum bed weight - Must be moved for visualization of upper arm complications or low femoral problems
RAMPART-IC	- Adjustable, motorized, lead-equivalent acrylic panel system - Adjustable lower lead shields	- Motorized system, allows ease of deployment and movement, with small footprint - Multiple access sites for catheterization	- System must be charged, which can take up to 10 h, but typically only once a year
ZeroGravity	- A full body 1.0 mm lead shield suspended from either a ceiling mounted monorail, swing arm, or wheeled floor unit - 0.5 mm acrylic face shield protects the head, eyes, and throat of the operator	- Operator mobility - Superior radiation shielding of head, neck, upper arms, and lower legs - Reduction in orthopedic strain - Ease of sterility - Adaptability to operator height	- Floor or ceiling space required for mounting mechanism - Cost of mounting mechanism
Cathpax AF Cabin	- Wheeled, semi-enclosed cabin using panels of 2 mm lead equivalent acrylic - Arm cutouts with a protective cuff	- Reduction in orthopedic strain - Ease of sterility - No installation costs	- Size of device limits use in tight spaces - Potentially limited application to left femoral vessels and left radial artery access - Limited adaptability to operator height - Visual impairment through device
Radiaction Medical	- “Plug and Play” accessory to the C-Arm - Flexible fins at the end of the device to reduce C-arm scatter - Proximity sensors present for customized to accommodate varying patient size	- Customizes fit to size and location of patient - Does not impede access sites	- Not yet approved for general market use - Still requires use of lead - Not compatible with biplane systems
Corindus CorPath 200	- Two major components: bedside unit and interventional cockpit	- High rate of device technical success in limited, small study - Remote operator shielding for robotic system	- Cost - Requires training for robotic-assisted coronary PCI - Limited to “simple” PCI - Still requires a “scrubbed in” staff member to help load/unload equipment
RADPAD	- Lead-free surgical drape made of barium and bismuth	- Simple addition to any setup - Cost effective - Easy to position	- Unclear benefits in operator radiation exposure at level of head

^*^This table illustrates each device’s key features as well as its unique benefits as well as pitfalls

## Partial room, large shielding system – The PROTEGO system

**Fig. 2:**
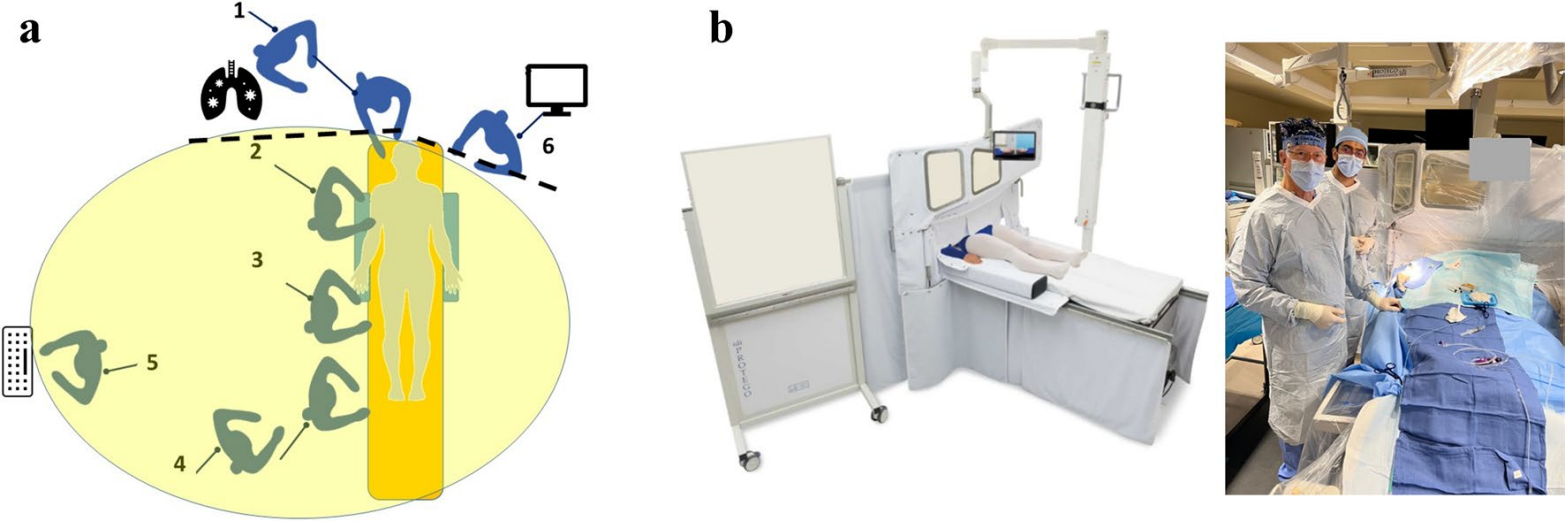
**A.** Diagram of locations which are exposed to radiation in the cath lab. Dotted lines represent the Protego shielding. The *yellow circle* is the umbrella of radiation protection. Compared to the pre-structural heart disease era, there are now personnel located closer to the x-ray tube for anesthesia and TEE operations. 1. respiratory therapist and anesthesiologist; 2. operator, 3. operator assistant, 4. circulating nurses, 5. recording technician/nurse, 6. echocardiographer. (Modified figure courtesy of Dr. Robert Wilson. From Cath Lab Digest, April 2023.) **B.** The PROTEGO system comprises of an upper shield mounted on a spring arm and attached to the table via magnets allowing for quick deployment and release. The lower shield is attached to the table and an operator side accessory shield to further reduce scatter. There are 2 patient visualization screens with a right and left camera to give the operator constant view of the patient throughout the procedure. There is also an arm board built in radiation drapes to allow for safe radial access. (*Reprinted with permission from Imaging Diagnostics, Inc.)*

The Protego Radiation Protection System (Image Diagnostics, Inc.) incorporates large shields over the table to reduce scatter to the operator’s side of the x-ray tube coupled with patient shield pads as well as lower table shields using both rigid shields and flexible radiation resistant drapes. The following encompasses the enclosure PROTEGO System (Fig. 2A). First is a strategically angulated upper shield above the table designed to passively accommodate unimpeded C-arm motion. This is mounted on a spring arm and attached to the table via magnets allowing for quick deployment and release. The next component is a lower shield attached to the table to reduce scatter downstream. An operator side accessory shield is present to further reduce scatter. The operator can always see the patient with patient visualization screens with left and right cameras. The PROTEGO system also includes a flexible radiation drip that extends from the patient’s lower abdomen to thigh to reduce scatter. In addition, an arm board with built in radiation drapes are present to allow for radial access. Lastly there are separate disposable radiation drapes that cover flexible and fixed components of the PROTEGO system. This shielding system casts an umbrella of protection for the operator and the circulating CCL staff (seen in Fig. 2B). There is early promising data demonstrating the PROTEGO system’s excellent radiation protection [3, 9, 10]. This has also been reproduced in a small study, achieving “zero” radiation exposure (RE) in two-thirds of cases using the PROTEGO system [11]. It is important to highlight that this system adds 100lbs reducing the maximum tolerable patient weight for the cath lab table. More advantages and limitations are highlighted in Table 1.

## RAMPART-IC

**Fig. 3:**
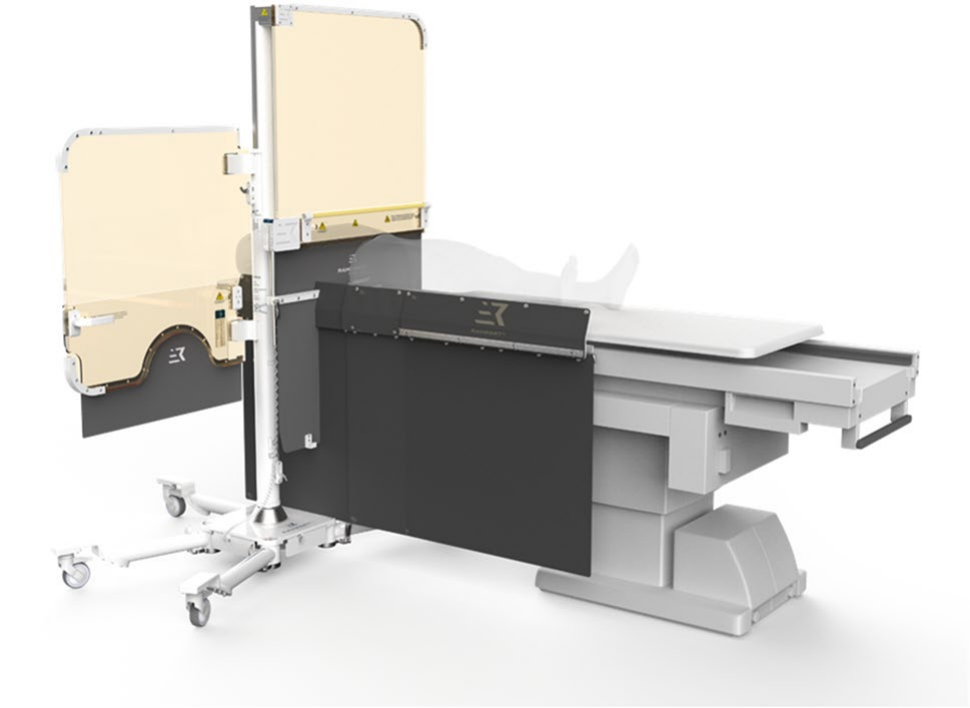
The RAMPART-IC is an adjustable, motorized, lead-equivalent acrylic panel system. Together with the adjustable shields, the sterile drape system accommodates radial, femoral, and pedal access catheterization. *(Reprinted with permission from RAMPART-IC LLC.)*

The RAMPART-IC is an adjustable, motorized, lead-equivalent acrylic panel system developed by RAMPART-IC LLC. On the company’s website, CEO and interventional cardiologist Dr. Bob Foster shares his personal story of work injuries associated lead aprons, leading to the development of a lead-free system that is now the combined Rampart M1128 shielding system and Rampart L138 table-mounted shield. Together with the adjustable shields, the sterile drape system accommodates radial, femoral, and pedal access catheterization (Fig. 3). Key studies demonstrate no difference in fluoroscopy time, dose-area product, or scatter radiation compared to traditional shielding, however overall lower total body radiation for all three positions of operators, including primary (fellow), secondary (attending), and tertiary (technician). [12] A limitation to this system highlighted in Table 1 is that it is battery operated and requires the system to be charged, which can take up to 10 h for a full charge. Fortunately, the system typically requires charging only once a year during regular use, and it also has the option to remain plugged in during use.

## Weight-Less, Suspended Lead Shielding – The Zero-Gravity [Biotronik]

**Fig. 4:**
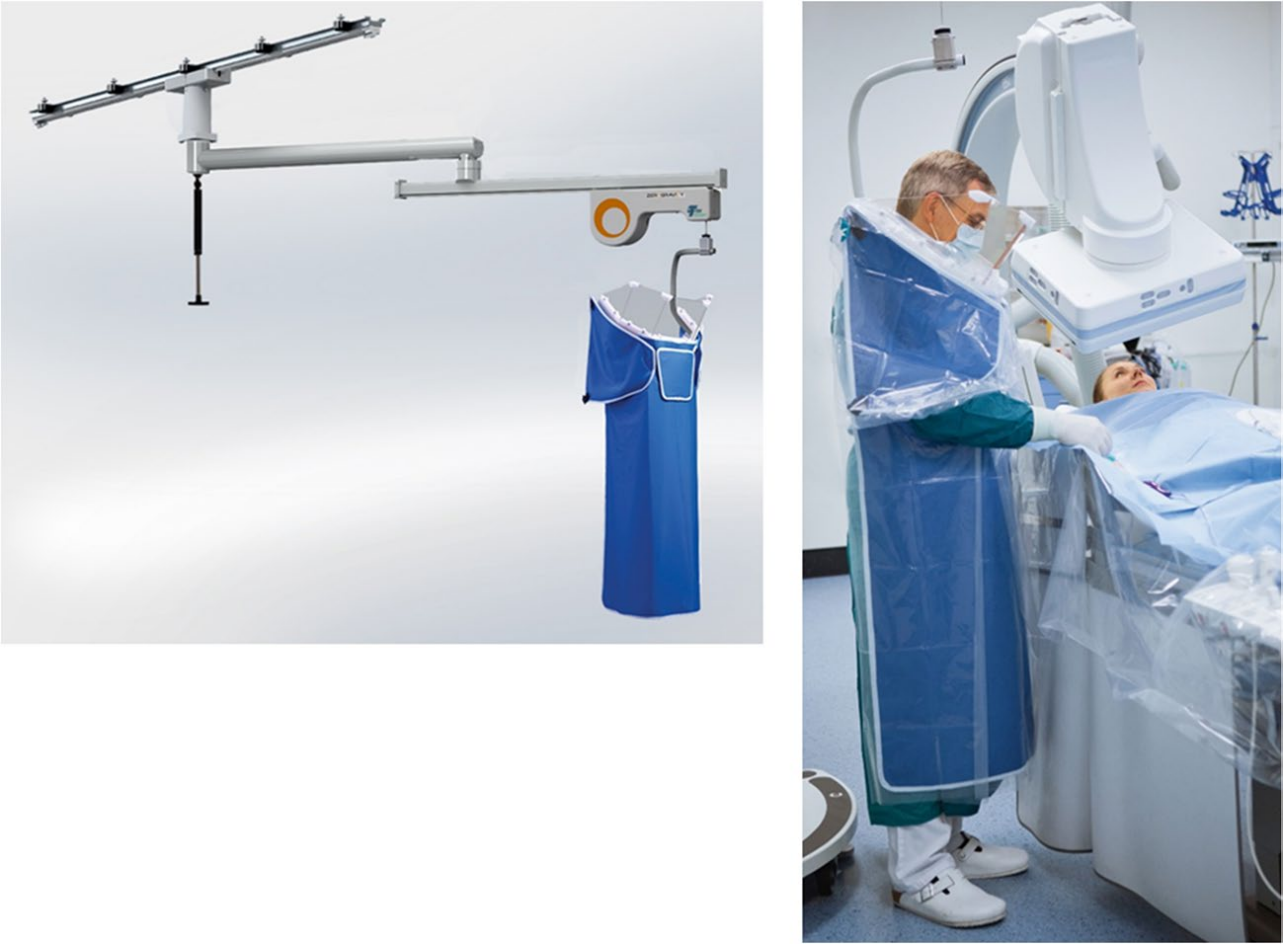
The ZeroGravity system is comprised of a full body 1.0 mm lead shield suspended from either a ceiling mounted monorail, swing arm, or wheeled floor unit. It is engaged to the operator magnetically via a lightweight vest. An additional 0.5 mm acrylic face shield protects the head, eyes, and throat of the operator while providing direct visualization of the surgical field. All traditional arterial and venous access sites are feasible, as well as full manipulation of the imaging arm. *(Reprinted with permission from BIOTRONIK, Inc.)*

Several systems have been designed with a focus on matching the operator mobility of wearable lead vests and aprons while providing equal or superior radiation protection. One such example is the ZeroGravity suspended personal radiation protection system. Originally developed by CFI Medical Solutions and later acquired by Biotronik, the ZeroGravity system (Fig. 4) is comprised of a full body 1.0 mm lead shield suspended from either a ceiling mounted monorail, swing arm, or wheeled floor unit. It is attached to the operator magnetically via a lightweight vest. An additional 0.5 mm acrylic face shield protects the head, eyes, and throat of the operator while providing direct visualization of the surgical field. Sterility is achieved using single use plastic coverings. This mounted adaptation of the traditional lead suit enables the operator to move freely around the operative table and suite while maintaining maximal radiation protection. All traditional arterial and venous access sites are feasible, as well as full manipulation of the imaging arm. Direct comparative studies of the ZeroGravity system against traditional lead and shields have demonstrated comparable radiation shielding in the torso with 87 -100% reduction in radiation dose to the head,eyes, neck, humerus, and tibia with ZeroGravity [13]. Additional studies have demonstrated lower total body [14] and head-level [15] radiation associated with use of the ZeroGravity system compared to traditional lead. In Savage et al., ergonomic survey of the testing operators reported superior comfort, less hassle, and less obstruction with use of ZeroGravity compared to traditional lead with shields. Further investigation is needed to comment on the prevention of chronic workplace orthopedic injuries when using a suspended lead system compared to conventional vests and aprons.

## Enclosed Operator Shielding – The CATHPAX AF Adjustable [for EP procedures only]

**Fig. 5:**
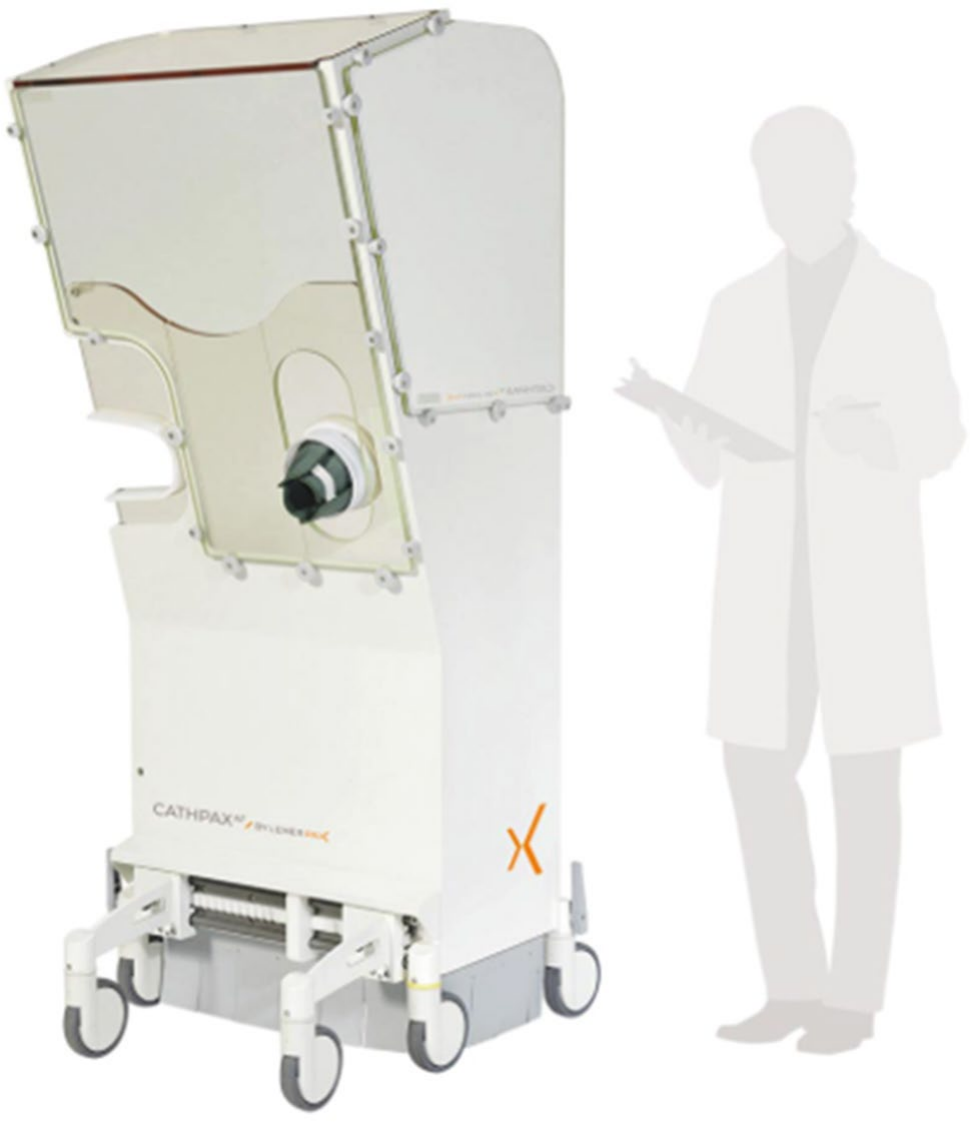
The Cathpax AF cabin is comprised of a wheeled, semi-enclosed cabin built using panels of 2 mm lead equivalent acrylic, weighing 210 kg and designed to accommodate adults up to 190 cm in height. Arm cutouts with a protective cuff are featured, as well as additional shielding on the left side of the operator closest to the ionizing source. The device is marketed to endovascular operators within interventional cardiology, electrophysiology, and other endovascular specialties utilizing fluoroscopy. Specific to interventional cardiology, the ability to reach across the field to access left radial or femoral artery sheaths may become challenging for certain operator-patient combinations. *(Reprinted with permission from Lemer Pax)*

Another product available on the market is the Cathpax AF cabin, developed by Dr. Michel Haissaguerre of the University of Bordeaux. The system (Fig. 5) is comprised of a wheeled, semi-enclosed cabin built using panels of 2 mm lead equivalent acrylic, weighing 210 kg and designed to accommodate adults up to 190 cm in height. Arm cutouts with a protective cuff is featured, as well as additional shielding on the left side of the operator closest to the ionizing source. Sterility is achieved using proprietary disposable drape. The device is marketed to endovascular operators within interventional cardiology, electrophysiology, and other endovascular specialties utilizing fluoroscopy. Compared to traditional wearable lead protection, the Cathpax AF cabin has been shown to reduce total body radiation by 74% in an early concept case series of 38 neurointerventional procedures described by Guersen et al. [16] In particular, the Cathpax AF cabin reduced head radiation exposure by 96% (0.14 mSv to < detection limit), thyroid by 78% (0.09 mSv to 0.02 mSv), and left sided body by 89% (0.45 mSV to 0.05 mSv). Differences in hand exposure were less pronounced when comparing traditional wearable lead to Cathpax AF, with right middle finger showing comparable exposure (0.06 mSv to 0.07 mSv) and left middle finger showing marginally improved protection in the cabin (0.22 mSv to 0.16 mSv). Eye protection with Cathpax AF was comparable to leaded goggles in this study. Ergonomic difficulties with the sizable nature of the cabin and its rigid design were described, with 38% of cases scoring unsatisfactory in the realm of reflections in the leaded panels, 24% of cases scoring unsatisfactory in accessibility to the field, and 14% of cases scoring unsatisfactory in hand mobility. Specific to interventional cardiology, the ability to reach across the field to access left radial or femoral artery sheaths may become challenging for certain operator-patient combinations. As Cathpax AF is designed to be one-size-fit-all, operators or patients at extremes of body stature may encounter ergonomic challenges.

## Remote Operator Shielding for Robotic System—Corindus CorPath 200 System

**Fig. 6:**
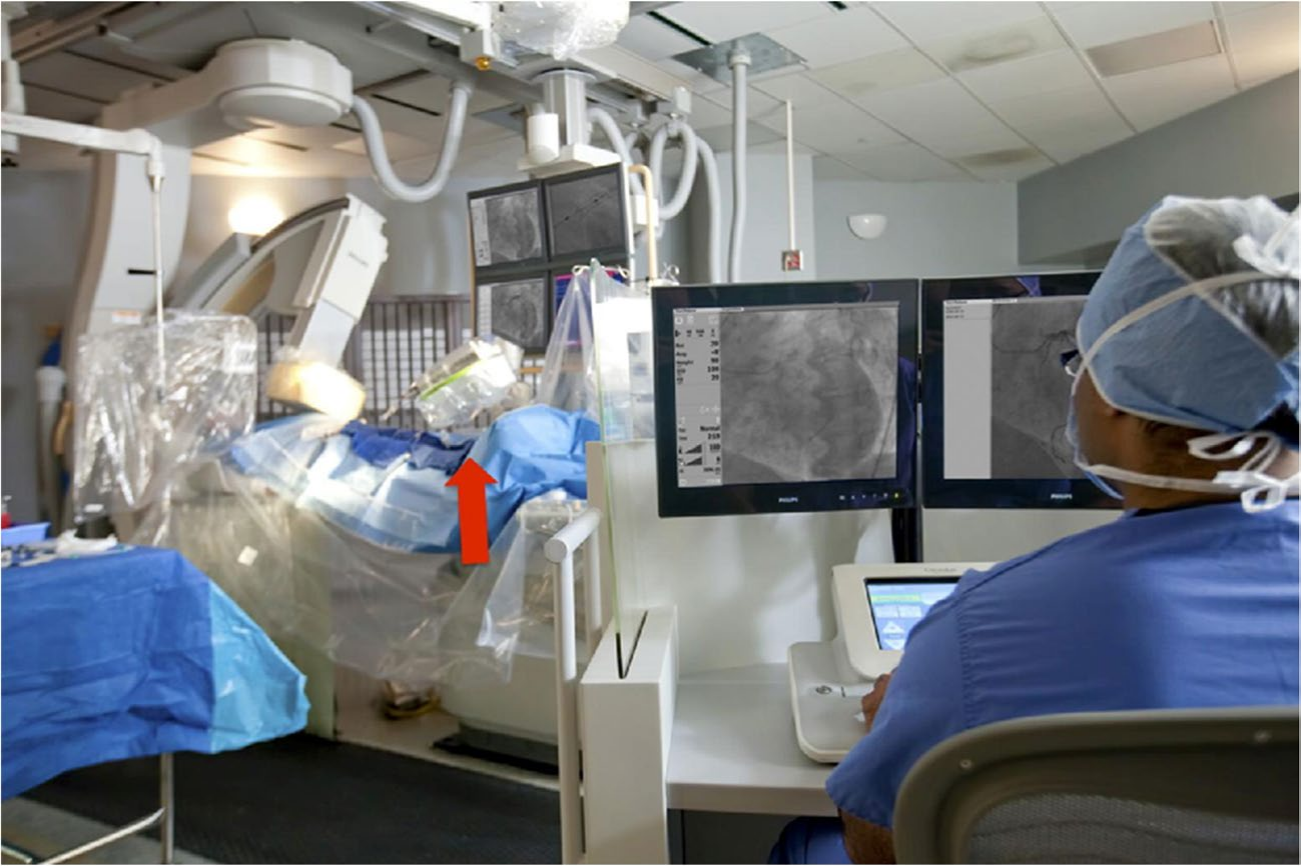
The Corindus CorPath 200 (indicated by the *red arrow*) was built to assist with coronary PCI and is comprised of 2 major components: the bedside unit [*left part of the image*] and the interventional cockpit [*right part of the image*]. The interventionalist is stationed at the cockpit and remotely performs the PCI using the joystick and touch-screen buttons of the CorPath 200’s console. The robotic cassette imposes axial and rotational forces on the intracoronary devices as it is loaded with interventional devices and is connected to the guide catheters. The CorPath 200 was developed to work with all 0.014-inch guidewires, rapid-exchange coronary angioplasty balloons, and stent delivery systems. (*Reprinted with permission from Philips)*

While devices and protective gear have been tested to address the occupational hazards associated with procedures in CCL, researchers in 2012 evaluated the safety as well as the clinical and technical success of robotic-assisted coronary PCI [17]. 164 patients underwent PCI at 9 sites with the robotic CorPath 200 System (Corindus Vascular Robotics, Natick, Massachusetts). Those with documented obstructive coronary artery disease (CAD) and evidence of ischemia were included. Those with complex disease were excluded [prior CABG, complex lesions requiring rotational atherectomy, intraluminal thrombus, severe tortuosity or calcification, bifurcation lesions, ostial lesions, and unprotected left main coronary arteries]. The CorPath 200 was built to assist with coronary PCI and is comprised of 2 major components: the bedside unit and the interventional cockpit. As expected, the interventionalist is stationed at the cockpit (Fig. 6) and remotely performs the PCI using the joystick and touch-screen buttons of the CorPath 200’s console. Cables running from the console to the robotic drive deliver commands which are relayed to a sterile cassette placed on top of the drive. The robotic cassette imposes axial and rotational forces on the intracoronary devices as it is loaded with interventional devices and is connected to the guide catheters. The CorPath 200 was developed to work with all 0.014-inch guidewires, rapid-exchange coronary angioplasty balloons, and stent delivery systems. Inside the cockpit, fluoroscopic, electrocardiographic, and hemodynamic images are visualized from a closer distance on the system's monitors. Both primary endpoints were successfully met. Clinical procedural success, which was defined as < 30% residual stenosis at the completion of the procedure without major adverse CV events within 30 days, was achieved in 160/164 patients. Additionally, there was a 95.2% reduction in median radiation exposure to the operators measured at the procedural table versus the interventional cockpit (20.6 vs 0.98 μGy, p < 0.0001). This study demonstrates that robotic assisted PCI can potentially used without affecting patient safety and procedural performance, at least for straightforward PCI. This device shows promise, however cost and further training for robotic assisted coronary PCI and possible delays in transitioning to a manual control during unforeseen procedural complications may be a limiting factor (Table 1). [17] When an unforeseen complication occurs and/or the primary operator would like to resume manual control, an “emergency stop” button located on the robotic console is pressed. Once this is pressed the operator has complete manual control and would need to enter the CCL including wearing lead and scrubbing in which can create delays in case of an emergency. One CCL staff member is required to be scrubbed and leaded to help load and unload equipment.

## X-ray Tube Shielding—Radiaction Medical

**Fig. 7:**
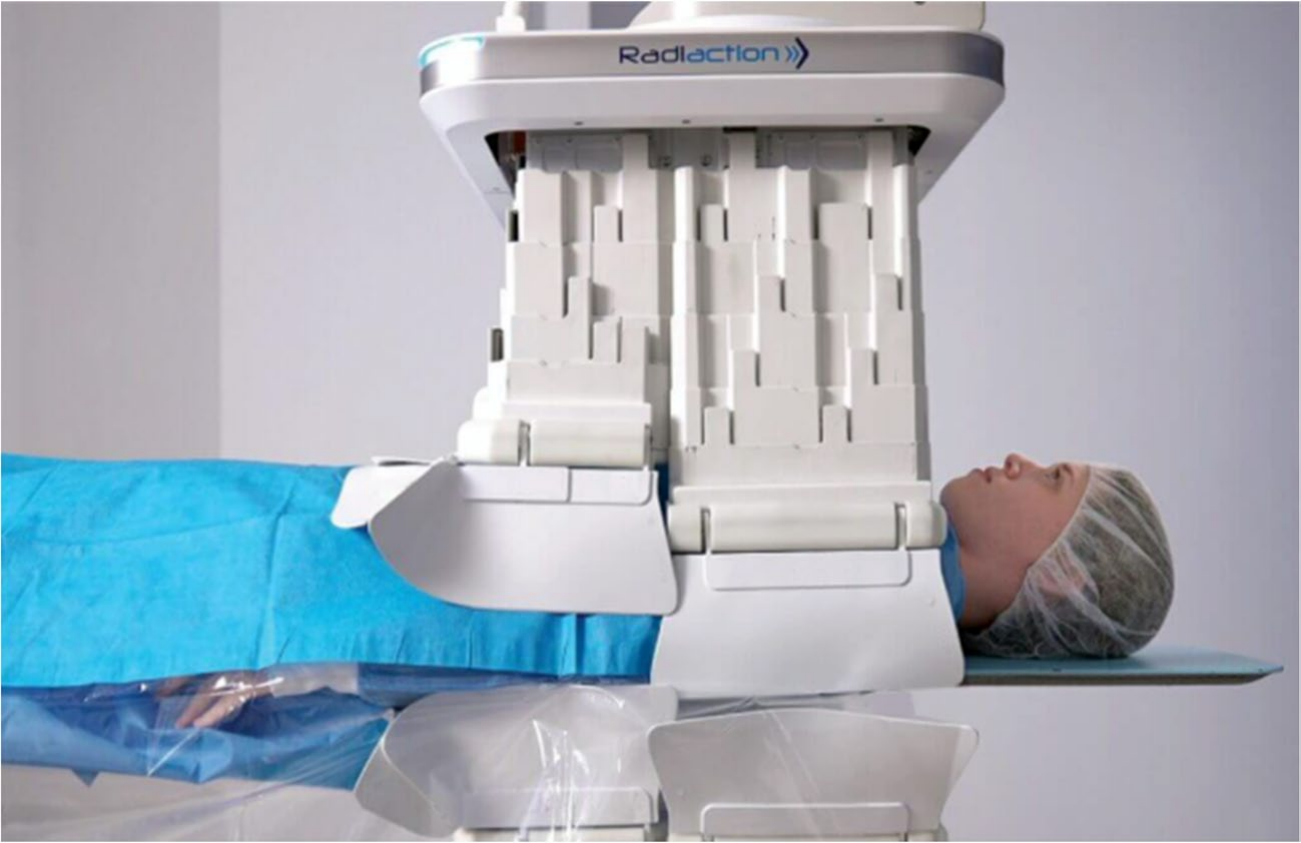
Radiaction Medical aids in reducing C-arm scatter by implanting a “plug and play” accessory to the C-arm, employing a “tunnel” mechanism with flexible fins at the end of the device to reduce C-arm scatter. The device has proximity sensors built into the device to allow for customized fit based on the size and location of each patient. (*Reprinted with permission from Radiaction Medical)*

Radiaction Medical is one of the newest concepts to aid in providing comprehensive radiation protection. This device aids in reducing C-arm scatter by implanting a “plug and play” accessory to the C-arm. The accessory (Fig. 7) implements a “tunnel” mechanism with flexible fins at the end of the device to reduce C-arm scatter. The device has proximity sensors built into the device to allow for customized fit based on the size and location of each patient. Conceptionally this product mirrors the 360-degree radiation scatter protection design of the Eggnest XR. This product is one of the newest concepts within the field of radiation protection, and a current small internal study show promising results with an over 80% reduction in radiation exposure among electrophysiology (EP) ablations and cardiovascular implantable electronic device (CIED) implantations [18]. Further advantages are highlighted in Table 1. This product is not currently on the market for approved use at this time.

## Disposable Adjunctive Lead Shielding Pads—RAPAD/NOPAD/SHAMPAD

**Fig. 8:**
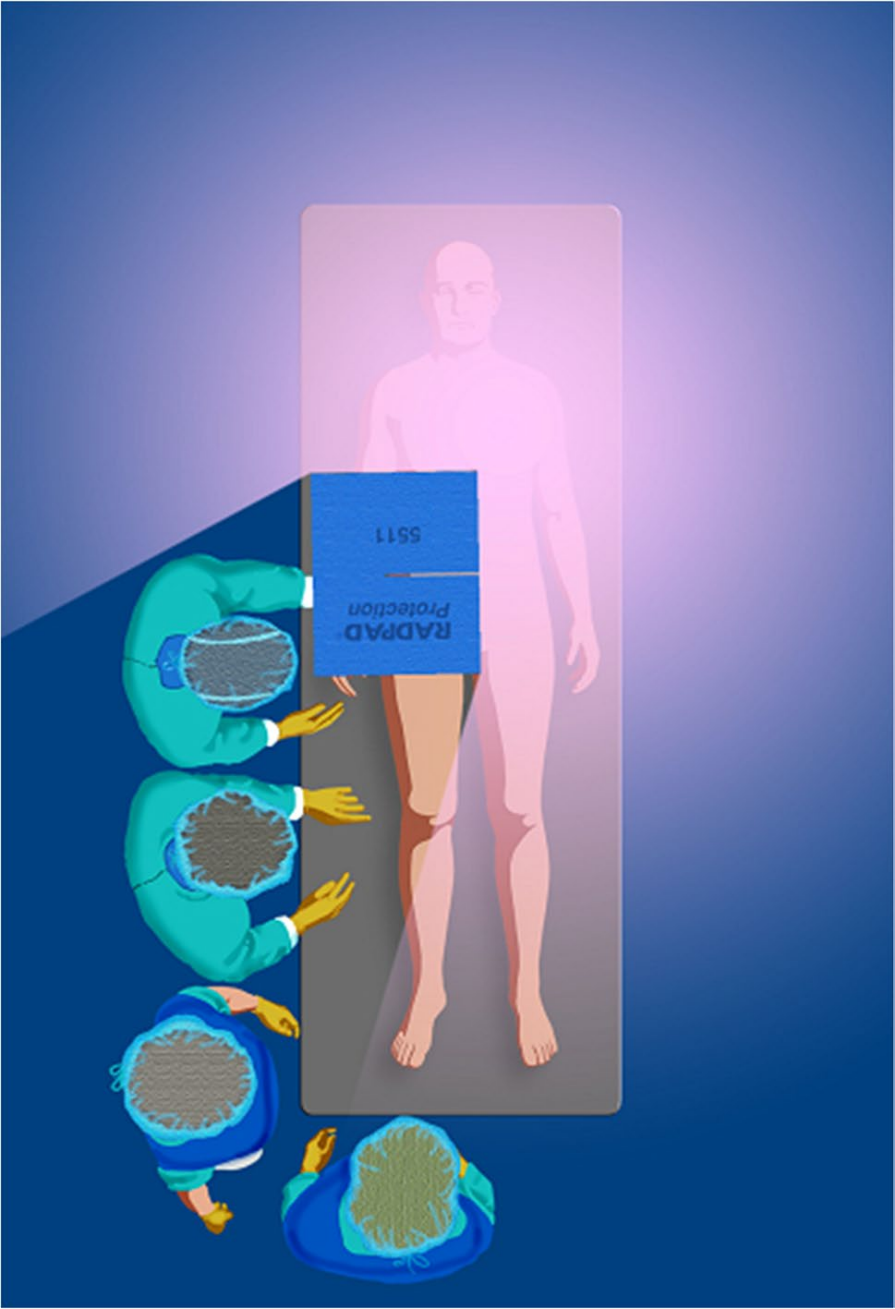
The RADPAD is a lead-free surgical drape made of barium and bismuth developed to be an environmentally friendly product that can effectively attenuate scatter radiation to protect physicians [*green*] and medical staff [*blue*]. The absorbing shield is typically placed flat on top of the patient [on table] in between the image intensifier and the primary operator. *(Reprinted with permission from RADPAD)*

The RADPAD is a lead-equivalent surgical drape made of barium and bismuth developed to be an environmentally friendly product that can effectively attenuate scatter radiation to protect physicians and medical staff. The absorbing shield is typically placed flat on top of the patient in between the image intensifier and the primary operator. (Fig. 8). RADPAD has shown significant reduction in mean total radiation exposure to the primary operator when undergoing transradial coronary angiography (282.8 ± 32.55 vs. 367.8 ± 105.4 μSv, P < 0.0001) [19]. A similar reduction is using RADPAD in transfemoral cases as well as complex PCI despite similar fluoroscopy times. [20, 21]. RADPAD has also been shown to significantly reduce mean primary operatory radiation dose during elective transcatheter aortic valve implantation. [22]

While the aforementioned studies investigated RADPADs efficacy when compared to conventional shielding methods, researchers in 2017 performed a sham-controlled, double-blind, all-corner, large randomized trial analyzing the efficacy of RADPAD when compared to conventional shielding measures (NOPAD) and a sham shield (SHAMPAD) [23]. Utilization of the RADPAD was associated with a 44% relative operator exposure reduction compared to the SHAMPAD (P < 0.001) and 20% reduction compared to the NOPAD. This study, the largest to investigate the impact of the shield, supports the RADPADs routine use in CCL. The RADPAD is an effective sterile disposable lead shielding pad that may one day be commonplace in CCL given the increasing complexity of procedures and the known risks from exposure to low-dose radiation.

## Conclusion

Innovative efforts to move towards a lead-apron free CCL continue to emerge. Some protection systems are already currently being utilized, and others are still in development. Each solution strives to provide maximum protection for staff while maintaining mobility for the operator to work freely. From radiation pads to entire operator enclosure devices, each protection system has its own unique advantages and limitations. In addition to employing novel shielding systems, radiation safety remains a principal duty of the operators to practice proven radiation reduction methods during procedures. Coupled with the innovative efforts described above, the lives of our patients and cath lab teams will be significantly improved. With the help and support of healthcare systems and administrators we can implement these innovative efforts to move closer to a lead-apron free CCL.

## Data Availability

No datasets were generated or analysed during the current study
